# 

**Published:** 1898-03

**Authors:** 


Secretary.

7½ South James St., Kansas City, Kan.

### PENNSYLVANIA STATE VETERINARY MEDICAL ASSOCIATION.

THE annual meeting of this Association will be held at the Veterinary Department of the University of Pennsylvania on Tuesday and Wednesday, March 8 and 9, 1898.

The local Committee of Arrangements, consisting of Drs. Kooker, Williams, Houldsworth, Gladfelter, Walter L. Hart, and Clarence J. Marshall, have completed all the preliminary arrangements, and a luncheon will be served each day by the veterinarians of Philadelphia to all those in attendance.

Dr. M. P. Ravenel, Bacteriologist to the State Veterinary Live-stock Sanitary Board will give an illustrated lecture from 7 to 8.30 on Tuesday evening. Dr. Leonard Pearson will tender a banquet to all visiting veterinarians and the Association members at 8.30 on Tuesday evening.

From 2 to 4 P.M. of the first day a series of operations will be performed by Drs. Adams, Zuill, Harger, and Hoffman, consisting of median neurotomy, tarsal tenotomy, ovariotomy, equine and canine, and tenotomy for stringhalt.

Among the papers to be read we note one by Dr. M. E. Conard, "What our Dairy-cattle Inherit;" Dr. H. B. Felton, "Pasteurization versus Purity;" Dr. J. Curtis Michener, "Feeding Animals;" Dr. M. P. Ravenel, "Milk from a Bacteriological Standpoint;" Dr. C. J. Marshall, "What we Ought to Know About Commercial Milk;" Dr. W. S. Phillips, "Tetanus;" Dr. W. P. Phipps, "Tetanus as I Have Found it in Chester County;" Dr. S. J. J. Harger, "Lesions of Spavin;" Dr. H. P. Keely, "Eversion of the Uterus in Cows."

Papers by Drs. Francis Bridges, Charles Bland, and J. F. Butterfield; subjects to be selected. Among the applicants for membership are: Drs. Morris W. Keck, Slatington; A. N. Lushington, William G. Shaw, and John A. Pearson, of Philadelphia.

All veterinarians of the State and visitors from other States will be very cordially welcomed to our meeting, and a generous invitation is extended.

JAMES B. RAYNER, V.S.,
President.

W. L. RHOADS, D.V.S.,
Corresponding Secretary.

---

"Imp. Great Tom," blind, but full of years as a great sire, died at Belle Meade Farm, Nashville, Tenn., on December 15th.

### PERSONALS.

Dr. J. C. McKenzie, of Rochester, N. Y., is lecturing to the horseshoers of that city and vicinity.

Dr. Jacob Wagner, of Tavistock, is a director of the Ontario Veterinary Medical Association, and is keenly interested in all matters pertaining to his profession throughout Ontario.

Dr. Leonard Pearson, State Veterinarian, addressed the Pennsylvania State Dairymen's Association, at Corry, in January.

Dr. M. E. Conard, of West Grove, addressed the Pennsylvania State Guernsey-Breeders' Association on "Parturient Apoplexy," in January.

Dr. Harry Walters, of Wilkesbarre, member of the Pennsylvania State Board of Veterinary Medical Examiners, was a visitor to Philadelphia in January.

Dr. Charles Dohan, of Darling, Pa., found much enjoyment following the hounds during the winter season.

Dr. H. D. Martien succeeds Dr. H. A. Christmann as Demonstrator of Anatomy at the Veterinary Department of the University of Pennsylvania.

Dr. M. Stalker, of Ames, Iowa, was a visitor to the East early in February, including a visit to Philadelphia and Washington.

Prof. James Law addressed the annual meeting of the New York State Breeders' Association in January.

Dr. A. T. Peters, of Lincoln, Neb., is the author of a recent bulletin for the Experiment Station of the "Blackwater State," on "Cornstalk-disease."

Dr. E. A. A. Grange lectured to the Detroit Horseshoers during the winter.

Dr. Martin has been elected Corresponding Secretary of the Toronto Master Horseshoers' Association.

The suit of Dr. J. H. Black, of St. Louis, Mo., against the Master- and Journeymen-Horseshoers for $20,000 for injury to character and loss of reputation, was on the docket for January.

Veterinarian E. B. Ackerman, of the Brooklyn Board of Health, was a visitor to the "City of Brotherly Love" in February.

Dr. F. W. Huntington, of Portland, has been elected Secretary of the Maine Mile-Track Association.

Dr. H. P. Eves, of Wilmington, Del., has been elected a member of the Executive Committee of the Wawaset Driving Association.

Dr. M. P. Ravenel, Bacteriologist of the Pennsylvania Live-Stock Sanitary Board, addressed the Farmers' Institute at Somerset, Pa., early in February.

State Veterinarian Pearson spent three days at the Somerset County, Pa., Farmers' Institute in February. "Stable Hygiene" and "Care of the Hoof" were among the topics he considered.

Dr. E. Merillat, of Chicago, is pursuing a course in human medicine at one of the medical colleges in Toledo, Ohio.

Mr. C. E. Stubbs is the special envoy selected by Secretary Wilson to visit Europe to interest foreign countries in the value of our horses for military and domestic purposes, and to better study the most desirable type of horse used for this purpose. Mr. Stubbs presented a paper on "The American Horse" at Denver, Col., in January, before the National Stock-growers' Convention.

Dr. S. E. Weber, after a short stay among his friends in Philadelphia, returned to Lancaster the latter part of January for a short rest, preparatory to entering the Philadelphia Orthopedic Hospital.

Dr. G. Howard Davidson, of Millbrook, is Vice-President for the second district of the New York State Agricultural Association.

Prof. A. Smith, of the Ontario Veterinary College, will conduct a course of instruction for the horseshoers of the city of Toronto, Canada.

Dr. Charles Gresswell, of Denver, has an excellent article in the December number of the *Colorado Medical Journal* on "Animal Diseases Communicable to Man," delivered at the annual meeting of the State Sanitary Convention.

Dr. D. McEachran, of Montreal, Canada, crossed the ocean for a short sojourn in Europe, in January.

Prof. A. Liautard, now in Paris, France, had as his guest early in February, Prof. D. McEachran, of Montreal, Canada, and together they visited, among other famous cities of "Sunny France," the famous watering-place, Nice.

Dr. James Henderson, Secretary of the Chicago Veterinary Society, will shortly leave the United States to accept a position in Scotland.

Dr. L. C. Campbell has returned to the post of Secretaryship of the Chicago Veterinary Society.

Dr. D. P. Frame, Food-Inspector of Colorado Springs, Col., has received an appointment under the Bureau of Animal Industry, and directed to report at Kansas City for duty.

Drs. Charles Williams, S. J. J. Harger, and John A. Pearson were interested witnesses at the third hearing of the Philadelphia Board of Health on the use of glass bottles and jars for the distribution of milk.

Drs. Austin Peters, of Boston, and Leonard Pearson and W. Horace Hoskins, of Philadelphia, as invited guests, participated in the discussion of municipal meat-inspection, considered by the New York County Veterinary Medical Association, on March 3d.

Dr. Samuel S. Buckley, of College Park, Md., spent a few days in Philadelphia in February.

Dr. Harry D. Entrikin, of Kennett Square, Pa., was elected a member of Town Council in February.

Dr. B. F. Bachman, of Strasburg, Pa., was one of the organizers of the local Jersey Cattle Club formed at Lancaster in February.

Drs. L. H. Howard, of Boston; W. Horace Hoskins, of Philadelphia; Otto Faust, of Poughkeepsie, and William Herbert Lowe, of Paterson, N. J., were New York City visitors early in February, attending a preliminary meeting connected with the closing exercises of the American Veterinary College.

Dr. Alexander Glass, of Philadelphia, was a visitor to the Westminster Kennel-Club Show at Madison Square Garden, in February.

Dr. B. M. Underhill, of Media, Pa., was a visitor to the JOURNAL office in February.

Messrs. John J. Repp, of the Class of '98, C. F. Keiter, of '99, and Thomas White, of the Class of 1900, were selected as representatives of the Veterinary Department in the Washington's Birthday exercises of the University of Pennsylvania in the Academy of Music.

Dr. George Coester, the non-graduate veterinarian appointed by Governor Pingree, of Michigan, as State Veterinarian, was found ineligible owing to his not possessing a diploma from any recognized college.

Dr. W. H. Ridge, of Trevose, Pa., was a visitor to the Western Sale-Stables, of Philadelphia, and the JOURNAL office in February.

Dr. Leonard Pearson, State Veterinarian of Pennsylvania, addressed the Farmers' Institute at Skippack in February.

City Veterinarian McNeil, of Pittsburg, entertained State Veterinarian Pearson in February.

Mr. Atwood B. Hoskins, of Glen Riddle, addressed the Students' Society of the Veterinary Department of the University of Pennsylvania upon the development of the various breeds of pigeons and their origin from the blue rock-dove. Some six or eight of the most distinct varieties were used to elucidate the wonderful changes produced in breeding.

Dr. Leonard Pearson is now a life-member of the Pennsylvania State Dairymen's Association.

Dr. George Jobson, of Franklin, Pa., will address the Pennsylvania Jersey-Cattle Club meeting the 17th of this month.

Dr. L. Breisacher, of Detroit, Mich., purchased the bay mare "Red Lawn" at the Fasig sale at Madison Square Garden in February.

